# Health Access Livelihood Framework Reveals Potential Barriers in the Control of Schistosomiasis in the Dongting Lake Area of Hunan Province, China

**DOI:** 10.1371/journal.pntd.0002350

**Published:** 2013-08-01

**Authors:** Julie Balen, Zhao-Chun Liu, Donald P. McManus, Giovanna Raso, Jürg Utzinger, Shui-Yuan Xiao, Dong-Bao Yu, Zheng-Yuan Zhao, Yue-Sheng Li

**Affiliations:** 1 Molecular Parasitology Laboratory, Division of Infectious Diseases, Queensland Institute of Medical Research, Brisbane, Australia; 2 School of Population Health, University of Queensland, Brisbane, Australia; 3 Institute of Global Health Innovation, Imperial College London, London, United Kingdom; 4 School of Public Health, Central South University, Changsha, People's Republic of China; 5 Département Environnement et Santé, Centre Suisse de Recherches Scientifiques en Côte d'Ivoire, Abidjan, Côte d'Ivoire; 6 Department of Epidemiology and Public Health, Swiss Tropical and Public Health Institute, Basel, Switzerland; 7 University of Basel, Basel, Switzerland; 8 Division of Health Education and Intervention, National Center for AIDS/STD Control and Prevention, Beijing, People's Republic of China; 9 Hunan Institute of Parasitic Diseases, Yueyang, People's Republic of China; Universidade Federal de Minas Gerais, Brazil

## Abstract

**Background:**

Access to health care is a major requirement in improving health and fostering socioeconomic development. In the People's Republic of China (P.R. China), considerable changes have occurred in the social, economic, and health systems with a shift from a centrally planned to a socialist market economy. This brought about great benefits and new challenges, particularly for vertical disease control programs, including schistosomiasis. We explored systemic barriers in access to equitable and effective control of schistosomiasis.

**Methodology:**

Between August 2002 and February 2003, 66 interviews with staff from anti-schistosomiasis control stations and six focus group discussions with health personnel were conducted in the Dongting Lake area, Hunan Province. Additionally, 79 patients with advanced schistosomiasis japonica were interviewed. The health access livelihood framework was utilized to examine availability, accessibility, affordability, adequacy, and acceptability of schistosomiasis-related health care.

**Principal Findings:**

We found sufficient availability of infrastructure and human resources at most control stations. Many patients with advanced schistosomiasis resided in non-endemic or moderately endemic areas, however, with poor accessibility to disease-specific knowledge and specialized health services. Moreover, none of the patients interviewed had any form of health insurance, resulting in high out-of-pocket expenditure or unaffordable care. Reports on the adequacy and acceptability of care were mixed.

**Conclusions/Significance:**

There is a need to strengthen health awareness and schistosomiasis surveillance in post-transmission control settings, as well as to reduce diagnostic and treatment costs. Further studies are needed to gain a multi-layered, in-depth understanding of remaining barriers, so that the ultimate goal of schistosomiasis elimination in P.R. China can be reached.

## Author Summary

China has made great strides toward reducing the burden of schistosomiasis, facilitated by sustained political commitment and a multi-faceted, integrated control strategy. The ultimate goal is disease elimination, which might be challenging due to high rates of re-infection, clusters of re-emergence, and growing health disparities. Market-oriented reforms and system-wide policies within the health care system offer new opportunities, but also entail challenges for the national schistosomiasis control program. Few studies have examined systemic barriers to equitable and effective schistosomiasis control in China. We explored the five core dimensions of access to health care, placing emphasis on schistosomiasis in the Dongting Lake area of Hunan Province. We collected and analyzed perspectives from staff working at local anti-schistosomiasis control stations and designated schistosomiasis hospitals, and from patients with advanced schistosomiasis. Our data suggest that a lack of affordability and high out-of-pocket expenditure posed a major barrier to the health care users, as did a lack of relevant health-information, and poorly accessible diagnostic and specialized surgical services. The lessons learned from this work are important in the design and development of disease control programs and entail key policy implications for schistosomiasis elimination.

## Introduction

Health disparities exist in all societies and recent data suggest that they might be widening [1]–[4]. Yet, many have argued that the existence of disparities in health status is unfair, should reasonable actions exist for their avoidance or mitigation [1], [4]–[10]. The concept of ‘fairness’ in health is related to (i) equality (the state of being equal) in health status; (ii) equality in health services; and (iii) equity (the quality of being fair and impartial) in health services [7]. Equality in health status may be impossible to achieve, given the vast array of behavioral, cultural, environmental, and genetic factors involved in generating each individuals' health outcome. Equality in health services is theoretically possible but, given inequalities in health status, this would strain finite resources and, hence, it is not desirable. Equity in health services, on the other hand, is characterized by universal access to essential health care and is key to achieving health for all [9], [11], [12]. Five inter-related dimensions of access to health care have been identified; availability, accessibility, affordability, adequacy, and acceptability of health-related goods, such as equipment and pharmaceuticals, and services, including prevention, diagnosis, and treatment [13]. Achieving health for all depends upon the context, policies, processes, institutions, and organizations that govern health-related goods and services, as well as the capacity among individuals, families, and communities to accrue assets and make use of them in times of need [10], [13]–[15].

The People's Republic of China (P.R. China) has undergone profound social, economic, political, and institutional transitions in the past decades as the centrally planned economy shifted to a socialist market economy. With the Chinese economy growing at an annual rate of 9–13% for the past 30 years [16], more than 210 million people were lifted out of poverty, benefiting from improvements in nutrition, drinking water, housing conditions, and health care. Within the health care system, major reforms included de-professionalization, de-centralization, de-regulation, commoditization, and economic restructuring [17], [18], creating both opportunities and challenges [19], [20]. Vertical disease control programs are considered particularly vulnerable to such changes and, indeed, for schistosomiasis control in P.R. China, substantial disparities may have emerged among the population [21], [22]. The Chinese government has been increasingly concerned with inequities and inequalities and, while the goals of generating economic growth and revenue remain, the creation of a peaceful and harmonious society is gaining prominence [23].

While the effects of the market-oriented reforms have frequently been analyzed with respect to rural primary health care in general, little is known regarding the specific case of schistosomiasis control [24]–[27]. The national schistosomiasis control program in P.R. China, launched in the mid-1950s, has reduced the prevalence of infection in humans by more than 90% through a multi-faceted integrated control approach, consisting of health education, environmental management, snail control, population screening, and large-scale treatment campaigns targeting humans and animal reservoir hosts, and the removal of bovine reservoirs and their replacement by mechanized agriculture [23], [27]–[29]. Much of this accomplishment can be attributed to strong and sustained political commitment and economic investment, including a 10-year World Bank loan project (WBLP) implemented between 1992 and 2001 [28], [30]–[33]. After termination of the WBLP, there were signs of re-emergence in some endemic areas, including an increase of advanced schistosomiasis japonica cases [28], [33], [34]–[37]. Among several reasons for the potential re-emergence of schistosomiasis, health systems reform has been identified as an important one [17], [37], [38].

Exploring systemic barriers to equitable and effective schistosomiasis control in P.R. China is of high importance. Here, we briefly review the history of the national schistosomiasis control program in Hunan Province, P.R. China. We then examine the five core dimensions of access to schistosomiasis-related health care in Hunan, using a framework proposed by Obrist and colleagues [13]. Emphasis is placed on perspectives from staff working at local anti-schistosomiasis control stations and designated schistosomiasis hospitals, and patients with advanced schistosomiasis japonica.

## Methods

### Ethics Statement

Ethical clearance for this study was obtained from the Medical Ethics Committee of Hunan Province and the Chinese Ministry of Health (MoH). The aims and objectives of the study were explained to each participant and written informed consent was obtained before beginning each interview and focus group discussion (FGD). All personal identifiers of the study notes and tapes were kept confidential and destroyed once the study was completed.

### Literature Review

Documents regarding policies and regulations associated with the prevention, diagnosis, and treatment of schistosomiasis were accessed from the following sources: (i) history of Hunan Province; (ii) history of health care in P.R. China; (iii) research on schistosomiasis control in Hunan Province; (iv) compilation of statistical data on schistosomiasis control in Hunan Province; (v) statistical data on schistosomiasis control in Hunan Province (1983–2001); (vi) atlas of schistosomiasis in Hunan Province; (vii) history of Hunan Institute of Parasitic Diseases; (viii) history of schistosomiasis control in Huarong County; and (ix) selected compilation of regulations on schistosomiasis control in Hunan Province. Government documents were accessed from (i) the Foreign Loan Office, Beijing; (ii) the Chinese MoH, Beijing; and (iii) the Hunan Provincial Office for Schistosomiasis Control. All unpublished data (‘grey literature’) were checked and validated through year-books of the Hunan Institute of Parasitic Diseases.

### Study Area and Sampling Population

A total of 145 key informant interviews and six FGDs were conducted in six schistosome-endemic counties in the Dongting Lake area, Hunan Province, between August 2002 and February 2003. These were Lixian and Hanshou in Changde City, Nanxian and Yuanjaing in Yiyang City, and Huarong and Yueyang in Yueyang City.

A mix of staff were selected through a purposeful sampling technique based on the value of their knowledge to the research to be undertaken; these included: (i) physicians or surgeons working in anti-schistosomiasis control stations who are acquainted with the diagnosis of advanced schistosomiasis; (ii) local staff involved in the administration and management of schistosomiasis control measures; and (iii) other schistosomiasis experts working at the Changde City Anti-Schistosomiasis Office and Institute, the Yiyang City Anti-Schistosomiasis Office and Institute, and Hunan Institute of Parasitic Diseases.

Patients with advanced schistosomiasis japonica were selected by convenience sampling according to registration lists and medical history records at the local anti-schistosomiasis control stations and designated hospitals taking care of patients with advanced schistosomiasis. Hospital records were used to trace the location of non-hospitalized individuals with advanced schistosomiasis. Interviewing of staff and patients was undertaken within the anti-schistosomiasis control stations or hospitals. Non-hospitalized individuals with advanced schistosomiasis were interviewed at home or in their village of residence.

### Interviews and FGDs

Interviews were undertaken face-to-face with each participant by researchers and interviewees and lasted between 30 and 90 min each. Demographic, occupational, and socioeconomic variables were filled in on the interview form according to the answers of each individual interviewee, while other information was tape-recorded and notes were taken, with the consent of the participant. Interview guides were prepared collectively by the co-authors and according to themes emerging from the published literature and prior experience and expertise. Staff interviews consisted of 16 closed and three open-ended questions, including views of the current approach to schistosomiasis control, opinions regarding the organization, management, and financing of schistosomiasis control, perceived technical quality, and responsiveness as well as potential barriers to sustainable control. Patient interviews included questions on schistosomiasis-related knowledge, attitudes, and practice (KAP), health-seeking behavior associated costs, experience of hospitalization, and patient satisfaction.

The FGDs guide was prepared collectively by the co-authors, the contents of which included the evolution of schistosomiasis control, the dynamics of the endemic status of advanced schistosomiasis, and future recommendations for sustained control of schistosomiasis. With the consent of the participants, the FGDs were tape-recorded, transcribed into Chinese, and later translated into English. Transcripts were complemented with notes taken during the FGDs. For consistency, all interviews and FGDs were undertaken by the same interviewer, who had received training in sociology, anthropology, and communication. In addition, the interviewer was native to the region and was familiar with local dialects, customs, and values.

### Statistical Analysis

Data were double entered, cross-checked, and validated. Quantitative data analyses were performed using SPSS version 16.0 (Illinois, USA). A temporal trend analysis was carried out to examine changes in the endemic status of acute and advanced schistosomiasis in Hunan Province between 1956 and 2001. Case detection, treatment, and cause-specific mortality rates were analyzed separately. Work space infrastructure, such as the number of anti-schistosomiasis control stations and institutes, and the number of beds within them, human resources, including full-time and temporary staff, and medical equipment were examined by a temporal trend analysis, as proxy variables for the availability of services.

Interviews and FGDs were examined by content analysis. We created a code list, categorized all interviews and FGDs according to the code list, and entered the data into a MaxQDA 2007 database (Marburg, Germany). Using the health access livelihood framework, we identified key concepts based upon the frequency with which they occurred within the database, and explored the relationships between them. Conceptual and relational analyses were conducted for explicit terms only; implicit terms were not examined. Demographic and socioeconomic variables were examined using χ^2^ test statistic.

## Results

### Historical Trends of Schistosomiasis in Hunan Province

**Figure 1:**
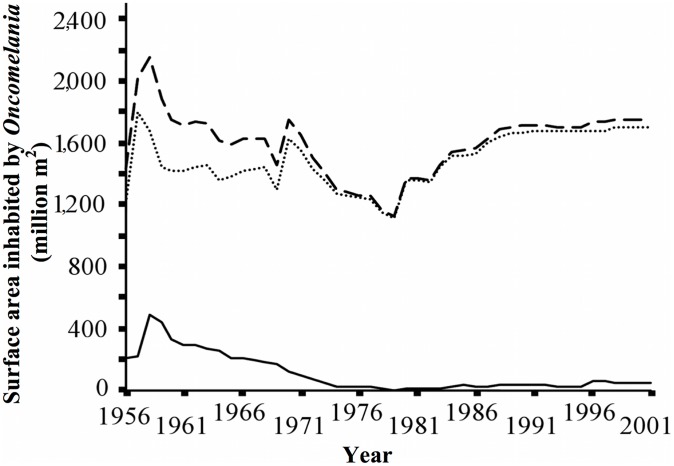
Estimated size of *Oncomelania* spp. snail habitats in Hunan Province, P.R. China, 1956–2001. Dashed line indicates total habitat size, dotted line indicates size inside embankments and filled line indicates that outside embankments.

Historical data suggested that the total area of land in the Dongting Lake area inhabited by *Oncomelania hupensis hupensis* (the intermediate host snail of *Schistosoma japonicum* in P.R. China), peaked at 2,159 million m^2^ in 1958. It gradually decreased to 1,756 million m^2^ in 1970 and to 1,121 million m^2^ in 1979. Thereafter, the snail-habitat increased. A similar trajectory was observed for the snail-populated area outside of lake embankments that were constructed around the Dongting Lake to provide additional farmland and protection from Yangtze River floods (Figure 1).

**Figure 2:**
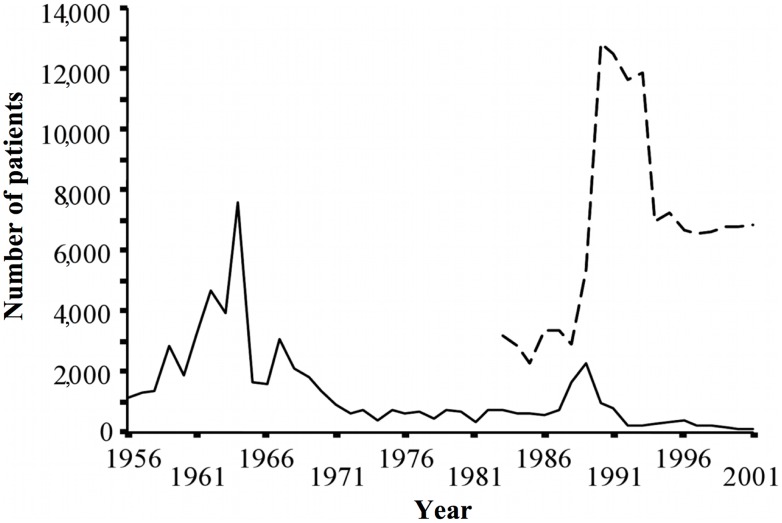
Estimated number of acute and advanced schistosomiasis japonica cases in Hunan Province, P.R. China, 1956–2001. Filled line indicates acute cases, dashed line indicates advanced cases.

The estimated number of schistosomiasis cases in Hunan Province gradually decreased and stabilized at approximately 225,000 cumulative cases in 1993. The number of patients with acute schistosomiasis japonica peaked in 1964 (7,572 cases). Twenty-five years later, in 1989, the number of acute cases showed more than a two-third reduction (2,259 cases) (Figure 2). With regard to the number of patients with advanced schistosomiasis japonica, no data were available prior to 1982. A first peak appeared in 1989 with 13,000 cases. Between the late 1990s and 2001, approximately 7,000 cases in total were recorded (Figure 2).

**Figure 3:**
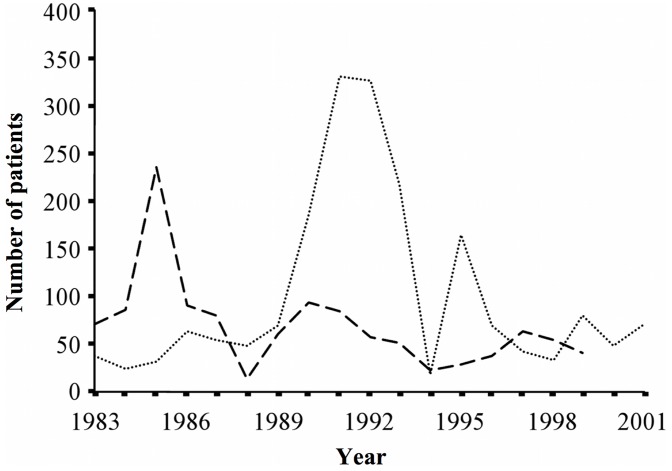
Estimated number of schistosomiasis japonica patients accessing specialized treatment services and schistosomiasis-related deaths. Dashed line indicates number of splenectomy operations performed, dotted line indicates the number of schistosomiasis-related deaths. Data from Hunan Province, P.R. China, 1983–2001.

The proportion of individuals with stunting and splenomegaly among the schistosome-infected population decreased over time. On the other hand, there was an apparent increase in liver cirrhosis and ascites, particularly among patients who concurrently had hepatitis. Splenectomy operations for advanced schistosomiasis patients were most common in the mid-1980s, with a subsequent declining trend (Figure 3). Mortality due to advanced schistosomiasis peaked in the early 1990s, decreasing and stabilizing thereafter (Figure 3).

### Operational Results and Characteristics of Interviewees

A total of 66 (62 or 95% male) staff from anti-schistosomiasis control stations and 79 (58 or 73% male) patients with advanced schistosomiasis were interviewed. Staff were mostly based at county level institutes (n = 35, 51%) with more than a third of them being senior staff members (n = 24, 37%). There were 26 physicians (40%), 21 surgeons (32%), eight directors or managers (12%), six policy makers (9%), and five consultants (8%). The age of the staff ranged from 30 to 72 years (mean = 49 years; standard deviation (SD) = 9 years). The interviewed staff had been employed for as long as 48 years (mean = 26 years; SD = 9 years) (Table 1).

**Table 1:** Demographic and occupational characteristics of key informants from Hunan Province, P.R. China.

Variables	Staff* (n = 66) (%)	Patients# (n = 79) (%)
Sex		
Male	62 (95.4)	58 (73.4)
Female	4 (4.6)	21 (26.6)
Age (years)		
20–39	5 (7.6)	4 (5.1)
40–59	53 (80.3)	40 (50.6)
≥60	8 (12.1)	35 (44.3)
Level of institute		
Provincial	6 (9.1)	n.a.
Prefectural	25 (37.9)	n.a.
County or below	35 (53.0)	n.a.
Rank or title		
Director	2 (3.0)	n.a.
Division/sub-division director	13 (19.7)	n.a.
Senior staff	24 (36.4)	n.a.
Intermediate staff	21 (31.8)	n.a.
Junior staff	6 (9.1)	n.a.
Job type		n.a.
Physician	26 (39.4)	n.a.
Surgeon	21 (31.8)	n.a.
Policy maker	6 (9.1)	n.a.
Manager	8 (12.1)	n.a.
Consultant	5 (7.6)	n.a.
Field work experience (years)		n.a.
10–19	12 (18.2)	n.a.
20–34	43 (65.2)	n.a.
≥35	11 (16.7)	n.a.
Education (years)		
0	0	22 (27.8)
1–6	0	46 (58.3)
7–9	50 (75.8)	7 (8.8)
10–12	16 (24.2)	4 (5.1)
Occupation		
Farmer/fisherman	n.a.	64 (81.0)
Civil servants	n.a.	4 (5.1)
Teachers	n.a.	6 (7.6)
Other	n.a.	5 (6.3)
Family type		
Nuclear	n.a.	52 (65.8)
Extended	n.a.	22 (27.9)
Living alone	n.a.	5 (6.3)

# Staff working at local anti-schistosomiasis control stations and designated schistosomiasis hospitals.

* Patients with advanced schistosomiasis.

n.a. = not applicable.

The age of patients ranged from 27 to 76 years (mean = 57 years; SD = 10 years). While 22 (28%) patients had no formal education, the remaining patients had between 1 and 15 years of schooling, often 6 years in total (n = 16, 20%). Most of the advanced schistosomiasis patients were farmers and/or fishermen (n = 64, 81%). Other professions included teachers (n = 6, 8%) and civil servants (n = 4, 5%), whereas five individuals (6%) did not specify their occupation. Most of the advanced schistosomiasis patients lived with their nuclear family (n = 52, 66%), while 22 (28%) lived with their extended family, and 5 (6%) lived alone (Table 1).

### Availability of Schistosomiasis Health Care Services

**Figure 4:**
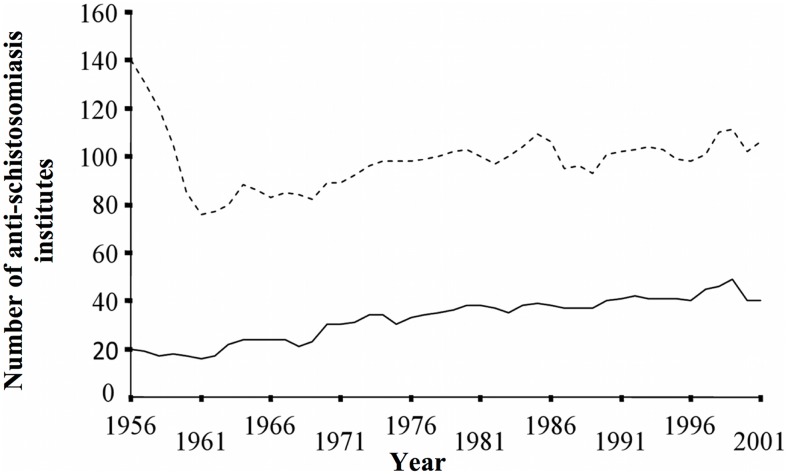
The number of professional and administrative anti-schistosomiasis institutes available in Hunan Province, P.R. China, 1956–2001. Dashed line indicates the number of professional institutes, filled line indicates the number of administrative institutes.

The number of professional anti-schistosomiasis institutes in Hunan almost halved in the first 5 years of operation; from 140 in 1956, to 76 in 1961. A slight increase to 106 institutes had occurred by 2001. The number of administrative institutes decreased from 20 in 1956, to 16 in 1961, but increased rapidly to a maximum of 49 in 1999 (Figure 4). The number of professional employees more than quadrupled from 959 in 1962, to 3,918 in 2001, and the number of administrative employees rose from a minimum of 65 in 1962, to a maximum of 347 in 1999. The number of employees per institute ranged from 12 to 37 (mean = 22; SD = 9) for the professional institutes and from 3 to 8 (mean = 6; SD = 1) for the administrative institutes.

**Figure 5:**
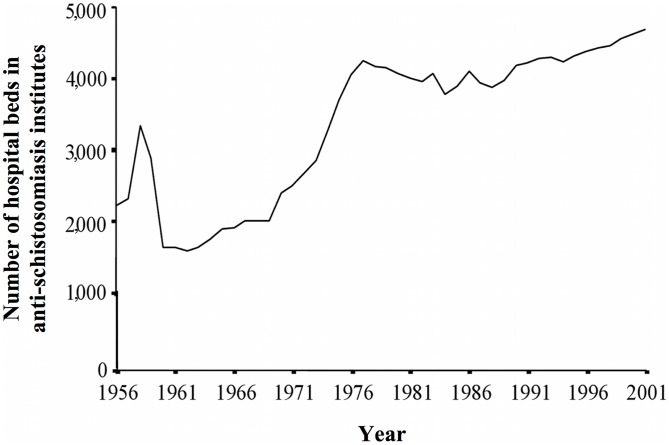
The number of hospital beds in anti-schistosomiasis institutes in Hunan Province, P.R. China, 1956–2001.

The number of beds available for schistosomiasis patients increased from 2,219 in 1956, to 3,346 in 1958, and then halved to 1,589 by 1962. A second peak of 4,256 beds occurred in 1977, which then decreased once more to 3,781 in 1984 and increased again steadily to the overall maximum of 4,697 beds in 2001 (Figure 5). The number of beds was highly significantly associated with the number of professional employees (r = 0.88, p<0.001) and the number of administrative employees (r = 0.90, p<0.001). Specific expertise of staff at anti-schistosomiasis control stations included the use of preventive measures such as snail control, surveillance methodology, health information, education, and communication (IEC) skills, as well as experience in clinical examination and chemotherapy procedures.

### Accessibility of Schistosomiasis Health Care Services

According to 30% of staff at anti-schistosomiasis control stations, most advanced schistosomiasis patients originated from non-endemic or only mildly endemic areas. A senior physician and schistosomiasis expert working at one anti-schistosomiasis control station explained it as follows:

*“Firstly, the current strategies for schistosomiasis control and the anti-schistosomiasis system focus on heavily endemic areas, while surveillance and control activities almost halt in mildly endemic and non-endemic areas, leading to the resurgence of advanced schistosomiasis. Secondly, people in mildly endemic and non-endemic areas, especially women and children, fall short on common knowledge regarding schistosomiasis control, increasing their contact with infested water, and hence also their risk of infection, and decreasing the likelihood that they will actively seek diagnosis and treatment of schistosomiasis.”*

Risk awareness and chemotherapy were reported to be high among residents in high-endemic areas, blocking progression of schistosomiasis from acute and chronic stages to the advanced stage. On the contrary, individuals, including health workers, from non-endemic or mildly endemic areas were said to often lack disease-specific knowledge vital for diagnosis and treatment. Sixty-five percent of anti-schistosomiasis control station staff mentioned this issue, which apparently was most critical for women and children. As a director for a county anti-schistosomiasis office explained:

*“Women are not often in contact with infested water, so they themselves don't pay much attention to it* [schistosomiasis]. *For example, when a woman went to visit her sister who is living in the endemic area, she got infected with schistosomiasis. But no-one paid much attention to it and she later developed serious schistosomiasis. A 9-year-old boy became infected when he played next to his aunt's boat in the lake, several times. There are no snails in the place he resides. His family took him to be examined in many hospitals, since he developed some serious symptoms. Eventually it was confirmed in a provincial hospital that he had late stage* [advanced] *schistosomiasis. Then he returned to the county hospital for splenectomy*.”

Over 80% of patients reported that if their symptoms were only mild or moderate, they would go to a nearby clinic or to the township level anti-schistosomiasis control stations to seek treatment. When their symptoms were very serious, however, they would go directly to the county level professional anti-schistosomiasis institutes to seek health care.

Furthermore, it was reported that splenectomy was only available at specialized anti-schistosomiasis institutes at the county level, or above, since lower level institutes lacked the equipment, facilities, and expertise to perform such operations. However, more than 90% of advanced schistosomiasis cases lived in the countryside.

### Affordability of Schistosomiasis Health Care Services

The mean annual per capita income in rural areas of Hunan Province in 2001 was CNY 2,299 (SD = 1,980), whilst in the families of the 79 patients suffering from advanced schistosomiasis, it was CNY 1,381 (SD = 1,060). The latter figure translates to approximately 60% of the rural average. The average medical cost of the 79 patients with advanced schistosomiasis was CNY 1,135, equating to 82% of the average annual income. Most of the medical expense (CNY 905, 80%) was directly due to schistosomiasis treatment. Medical spending was 12-fold higher among the advanced schistosomiasis patients interviewed than the per capita average (CNY 96) in the rural areas of Hunan Province in 2001. None of the patients had any form of health insurance and all expenses were out-of-pocket payments. Over half of the patients reported being unable to partake in strenuous farming activities due to their disease, but that they were also unable to cover the high costs of treatment and hospitalization:

*“If it's possible, of course I want to be treated, but I have no means since I have no money.”*

*“If you have money, you will use it to challenge schistosomiasis, and if you don't have money, you will use your life to pay for it.”*

Expenditures were highest for inpatient treatment. Seventy percent of patients reported preferring outpatient treatment and buying medicine at the local chemist to inpatient systematic hospitalization and treatment. Similarly, 65% of the patients reported that they would ask to be discharged from the hospital or schistosomiasis station once symptoms improved only slightly, due to a lack of financial resources. Furthermore, 30% of patients delayed seeking professional medical care, turning instead to cheaper forms of traditional medicine.

In 1994, the local government of Hunan Province established the Hunan Provincial Foundation for Treatment of Advanced Schistosomiasis. The aim of the foundation is to provide subsidies during hospitalization; however, this was only applicable to patients who live in the countryside or those who are extremely poor with severe signs and symptoms of advanced schistosomiasis. The amount of money provided depended on the severity of disease and on the availability of resources within the foundation, but usually varied between CNY 200 and CNY 500 per person per year. One out of five patients had been eligible to apply for the subsidy, which covered part of their total medical costs.

### Adequacy of Schistosomiasis Health Care Services

Ten percent of staff occasionally referred to gaps in the organization and management of human resources, with a viewpoint that a lack of thorough training, together with limited biomedical knowledge, resulted in poor quality of service provision. This was expressed by a director of a local anti-schistosomiasis control station and a surgeon, respectively, as follows:

*“More and more people enter into anti-schistosomiasis system, but only a few get the professional training.”*

*“Schistosomiasis control services were provided, but often without a bio-medical background and thorough training on schistosomiasis prevention and control.”*

A county level deputy-director expressed regret that, whereas in the past the central government allocated significant financial resources to subsidize the schistosomiasis control program, this was no longer the case and counties had to provide the largest financial share for control. The same key informant further emphasized that schistosomiasis-endemic areas were mostly located in economically underdeveloped areas and that the local governments of these regions had limited financial capacities:

*“In the 1970s, schistosomiasis control was emphasized and performed vigorously. But now the work is becoming more difficult. Although capital investment seems to have increased a lot, it has actually decreased because of a rise in prices, increases in the number of personnel and the costs of prevention and treatment.”*

### Acceptability of Schistosomiasis Health Care Services

Seventy-five percent of patients interviewed had known for a decade about their disease. They had acquired much knowledge in diagnosis and treatment through their personal experience and were often able to correctly identify modes of infection, prevention, and treatment. Fourty-five percent indicated that advanced schistosomiasis severely affected their health and that once they were diagnosed, they actively sought treatment. They believed that it was possible to cure the disease using modern scientific means and technology. However, the other 55% were more pessimistic about the disease. One 35-year-old male patient said:

*“The worst thing that ever happened to me is that I got the disease* [advanced schistosomiasis]. *It is my fate, and I can't beat schistosomiasis.”*

Patients who had considerable experiences of hospitalization in the local anti-schistosomiasis control stations and in general hospitals had a favorable impression of the services provided. For example, most felt that the doctors and staff were friendly and treated them fairly. However, 30% of the interviewees also said that they paid the bill without being able to assess the fairness of the charges. Mostly, they reported that when compared with the general hospital, the professional competence of the anti-schistosomiasis doctors and nurses was poorer, as were ward conditions and the equipment used. A 69-year-old male patient stated:

*“The service attitude at the anti-schistosomiasis hospital is reasonable but their techniques make us feel uneasy.”*

However, being hospitalized in an anti-schistosomiasis control station was cheaper and it provided the possibility of being subsidized.

## Discussion

This study provides a comprehensive analysis of the performance of a disease control program at the sub-national level. Within P.R. China, in particular, this is an important unit of analysis due to a highly decentralized fiscal system. We applied the health access livelihood framework [13] to the Chinese schistosomiasis control setting, using a mixed-methods approach with both quantitative and qualitative data, including historical records, key-informant semi-structured interviews, and FGDs. The results indicate that unaffordable diagnostic and treatment costs, relative to household spending capacity, posed a major barrier in access to schistosomiasis-related health care in Hunan Province. Although sufficient infrastructure and human resources were available at local control stations, relevant health information, diagnostics, and specialized surgical services were poorly accessible to patients residing in non-endemic or low-risk areas. Moreover, staff implied that resource organization and management were somewhat inadequate, although patients felt that care was acceptable and that doctors and other health personnel were friendly and treated them well. The lessons learned from the current research have several important policy implications, particularly in view of P.R. China's goal to eliminate schistosomiasis.

Over the past 60 years, P.R. China has demonstrated major achievements in schistosomiasis control [27]–[29], [39]–[44] and the provincial level historical trends in our dataset indicate that this is also the case for Hunan Province. Nonetheless, the data in Figure 1 indicate that in 2001 there was still an increased schistosomiasis risk as a result of marginal temporal changes in the size of the snail habitat area inside the embankments and their effect on local schistosomiasis endemicity [45]. The nationwide control program was successful from its inception, although less so during the economic, political, and social upheaval of the 1960s [19], [46], [47]. Between 1983 and 1988 schistosomiasis re-emerged considerably, possibly as a result of changes in the rural economy and a severe reduction in finances directed toward schistosomiasis control [48]. The Chinese Government continued to pay attention to the control of acute schistosome infections, this being a sensitive issue. Thus, acute cases were kept at a relatively low level. Ultrasonography was introduced to measure morbidity caused by schistosomiasis during the middle of the 1980s. The resulting improvements in diagnosis led to more advanced cases being identified and reported, indicated by the sharp peak shown in Figure 2. Following an outbreak of acute schistosomiasis in 1989 in Wuhan, Hubei Province [49], [50], a major metropolitan area neighboring Hunan Province, a decade-long robust targeted control ensued. This was supported by an overall investment of US$ 82 million from the Chinese government, complemented by a further US$ 71 million through the WBLP [29], [32], [51]. More recently, the national control program has been further strengthened and prioritized, as P.R. China aims to move toward schistosomiasis elimination [23], [43], [52], [53].

Despite clear accomplishments, the data in Figure 2 suggest that, while acute and advanced cases of schistosomiasis declined considerably over time, both disease types remained of public health importance. This may be a result of substantial changes in human and animal population density connected to large water management projects in the Dongting Lake area [45], as well as daily water contact patterns of migrant farmers and fishermen. Whilst snail and animal reservoir host infection rates may have decreased as a result of large-scale chemotherapy, they remained important contributors towards the schistosomiasis transmission cycle.

In accordance with other studies [54], [55], we found that patients with advanced schistosomiasis japonica were, on average, above 40 years, with lower levels of general, and health-specific, education. We also found a growing trend of advanced schistosomiasis japonica among the young and/or female populations, especially those originating from low-endemic areas. This is likely to be due to a lack of awareness of key risk factors, such as contact with infested water, as well as the presence of non-specific symptoms which do not trigger the appropriate treatment-seeking behavior [56]–[59]. Moreover, populations from non- or low-endemic areas who relocate to endemic areas are at an increased risk of infection because of a lack of acquired immunity (i.e., persons never exposed to schistosomiasis) or diminished immunity (i.e., persons formerly exposed) [60]. In the Dongting Lake area, several thousands of people have migrated from non-endemic areas upstream of the Three Gorges Dam, and the system currently in place may fall short on meeting the distinct needs of such a mobile population, posing heightened risks of re-emergence [43]. We advocate enhancing disease control and surveillance in less endemic areas, and strengthening health education and awareness among residents in all areas.

Poverty is a key social determinant of schistosomiasis [24]–[26], [38], [41], [42], [61]. We found that patients with advanced schistosomiasis were among the poorest individuals in the community. High medical expenditures accounted for a considerable proportion of their annual income and a majority of the patients interviewed reported having no form of social security. In the mid-1950s, a collectivism-based rural health insurance system known as ‘co-operative medical system’ (CMS) was established in P.R. China [62], covering the cost of medical care, including the management of advanced schistosomiasis cases [63]. However, as the CMS collapsed at the end of the 1970s, the provision of schistosomiasis-related health care altered from free services to fee-for-service (i.e., mostly out-of-pocket) for all except government employees and state-run factory workers, and around 90% of the rural population were left uninsured. The Chinese government has recently recognized the need to increase health spending and promote new health insurance schemes such as the ‘new co-operative medical system’ (NCMS), which focuses on reducing the risk of catastrophic costs of healthcare [64], [65]. There are concerns, however, that this may be less effective in rural areas, where a majority of the population resides [66]. Furthermore, a series of regulations were implemented by the Chinese government to alleviate the financial burden of advanced schistosomiasis in endemic areas, but these regulations do not take into account cases in non-endemic areas [67]. High treatment costs prevented 35% of patients interviewed from seeking professional medical care, whilst others may have been forced to sell productive assets or may have become indebted and subjected to high short-term interest rates, in order to finance their healthcare. These strategies for coping impact people's livelihoods, increase their vulnerability, and reduce their capacity to negotiate their way out of poverty. If many households experience similar problems, this may have a negative effect on a community's capacity to overcome such shocks. In order to establish a comprehensive social security system applicable to the entire population, efforts should be made to develop feasible, practical, and sustainable health insurance and social support systems with special attention to the most vulnerable and marginalized groups, such as remote and impoverished patients suffering from advanced schistosomiasis.

We found that the availability of physical resources (e.g., number of hospital beds), and human resources (e.g., number of trained health personnel) increased over time although the prevalence of *S. japonicum* decreased. Broadening control efforts to include other parasitic diseases may maximize the benefits of available resources, improve efficiency and resource allocation, and increase cost-effectiveness, particularly in light of the incorporation of novel technologies such as geographical information systems (GIS) and remote sensing for disease surveillance in remote areas [41], [43], [52], [53], [66], [68], [69]. This expanded profile of parasites may include soil-transmitted helminthiasis and intestinal protozoa infections, which are highly prevalent in many areas of P.R. China [25], [70]. Historical observations suggest that the availability of schistosomiasis-related health care is largely due to the Chinese governments' perceived public health importance of schistosomiasis, which was placed high in the political agenda and treated as a public or semi-public good. In view of recent market-oriented health-care reforms, the relative roles of the government and market in health services for P.R. China should be re-assessed [17], [21], [22].

According to a majority of interviewees' perceptions, there were no major barriers in terms of adequacy and acceptability of schistosomiasis control, although several patients and staff expressed a need for improved technical expertise and efficiency at the grass-roots level. Studies in various country settings have found positive associations between health service provider-user relationships and user satisfaction, with a lack of clear and effective communication being a key undermining factor in provider-user interactions, negatively affecting health seeking behavior through a lack of trust [71], [72]. In P.R. China, a review of general health services delivery reported patient complaints about unclear information and communication regarding the services received [73]. In our study, a small number of patients showed signs of distrust of doctors with regard to payment and reimbursement policies. In addition, some staff claimed that providers may recommend more expensive tests than necessary, suggesting that considerations other than clinical needs are influencing their provision. These results highlight the need for increased staff training, improved communication, and an appropriate regulatory framework which redefines more clearly and transparently the responsibilities of schistosomiasis control staff particularly in light of comprehensive integrated control that includes cross-sectoral collaboration and co-operation [23], [52], [74]–[75].

The study was subject to several limitations. First, its small-scale and exploratory design precludes generalization at the provincial or national level for P.R. China. The study area is, however, a major endemic focus, epidemiologically representative of schistosomiasis control in Hunan Province and other marsh and lake regions in southern P.R. China such as the Poyang Lake area in Jiangxi Province. Second, the qualitative data are subject to a number of biases, including recall bias, partial inclinations according to the interviewees' judgment of what the interviewers wished to hear, or by reluctance to talk about sensitive issues. There is also a possibility of gender bias due to the under-representation of female key informants. Nevertheless, the study aimed to highlight key issues and interests among providers and recipients of schistosomiasis control activities, and rigorous measures were taken throughout to reduce such biases. Third, the data collection was undertaken more than 10 years ago, and hence it is conceivable that people's perceptions have changed and schistosomiasis control services have further adapted to the changing epidemiology and control emphasis of the national program. However, advanced schistosomiasis is a chronic condition with people suffering from it many years after schistosome transmission has been interrupted [76]. We have now planned a similar study to determine whether disease-specific knowledge, specialized health care services, and rural health insurance have indeed improved.

In this study we illustrate the importance of taking a systemic approach to evaluation that links utilization and provision of health services with the wider context of policy making. According to Tang et al. [20] three major inter-related processes have emerged concurrently to create “a perfect storm” for health care in P.R. China. These are: (i) market failures and insufficient government stewardship; (ii) inequities in the social determinants of health; and (iii) the erosion of public perceptions of fairness and trust of the health-care system [20]. Our study has identified areas that require action within Hunan Province, and the results gained from applying the health-access livelihood framework indicated that, from the above three points, the second may be the most vital component for the control of schistosomiasis, followed closely by the first and the third. The findings are of particular relevance now that P.R. China's goal is to eliminate schistosomiasis and, even if elimination has been reached, they are of importance for post-transmission schistosomiasis [76]. This study also highlights future research needs in order to generate evidence for design and development of disease control programs.
